# The S-Phase Cyclin Clb5 Promotes rRNA Gene (rDNA) Stability by Maintaining Replication Initiation Efficiency in rDNA

**DOI:** 10.1128/MCB.00324-20

**Published:** 2021-04-22

**Authors:** Mayuko Goto, Mariko Sasaki, Takehiko Kobayashi

**Affiliations:** a Institute for Quantitative Biosciences, University of Tokyo, Tokyo, Japan; b Department of Biological Sciences, Graduate School of Science, University of Tokyo, Tokyo, Japan; c Collaborative Research Institute for Innovative Microbiology, University of Tokyo, Tokyo, Japan

**Keywords:** Clb5, ribosomal RNA gene, replication fork arrest, genome instability, replication origin

## Abstract

Regulation of replication origins is important for complete duplication of the genome, but the effect of origin activation on the cellular response to replication stress is poorly understood. The budding yeast rRNA gene (rDNA) forms tandem repeats and undergoes replication fork arrest at the replication fork barrier (RFB), inducing DNA double-strand breaks (DSBs) and genome instability accompanied by copy number alterations. Here, we demonstrate that the S-phase cyclin Clb5 promotes rDNA stability. Absence of Clb5 led to reduced efficiency of replication initiation in rDNA but had little effect on the number of replication forks arrested at the RFB, suggesting that arrival of the converging fork is delayed and forks are more stably arrested at the RFB. Deletion of *CLB5* affected neither DSB formation nor its repair at the RFB but led to homologous recombination-dependent rDNA instability. Therefore, arrested forks at the RFB may be subject to DSB-independent, recombination-dependent rDNA instability. The rDNA instability in *clb5Δ* was not completely suppressed by the absence of Fob1, which is responsible for fork arrest at the RFB. Thus, Clb5 establishes the proper interval for active replication origins and shortens the travel distance for DNA polymerases, which may reduce Fob1-independent DNA damage.

## Abstract (precis)

Regulation of replication origins is important for complete duplication of the genome, but the effect of origin activation on the cellular response to replication stress is poorly understood. The budding yeast rRNA gene (rDNA) forms tandem repeats and undergoes replication fork arrest at the replication fork barrier (RFB), inducing DNA double-strand breaks (DSBs) and genome instability accompanied by copy number alterations.

## INTRODUCTION

Precise duplication of the genome is crucial for maintaining genome integrity. However, DNA replication is constantly challenged by exogenous and endogenous stresses (1, 2). Obstacles such as DNA damage and tightly bound proteins cause the replication fork to stall. Stalled forks resume DNA replication when the replication stress is relieved or complete replication when a converging fork arrives (3). Failure to properly resolve a stalled fork results in chromosome rearrangements, such as sequence deletion, duplication, or inversion, and chromosome translocations, which are hallmarks of cancer cells, cause human genomic disorders, and influence genome diversity (2, 4).

**FIG 1:**
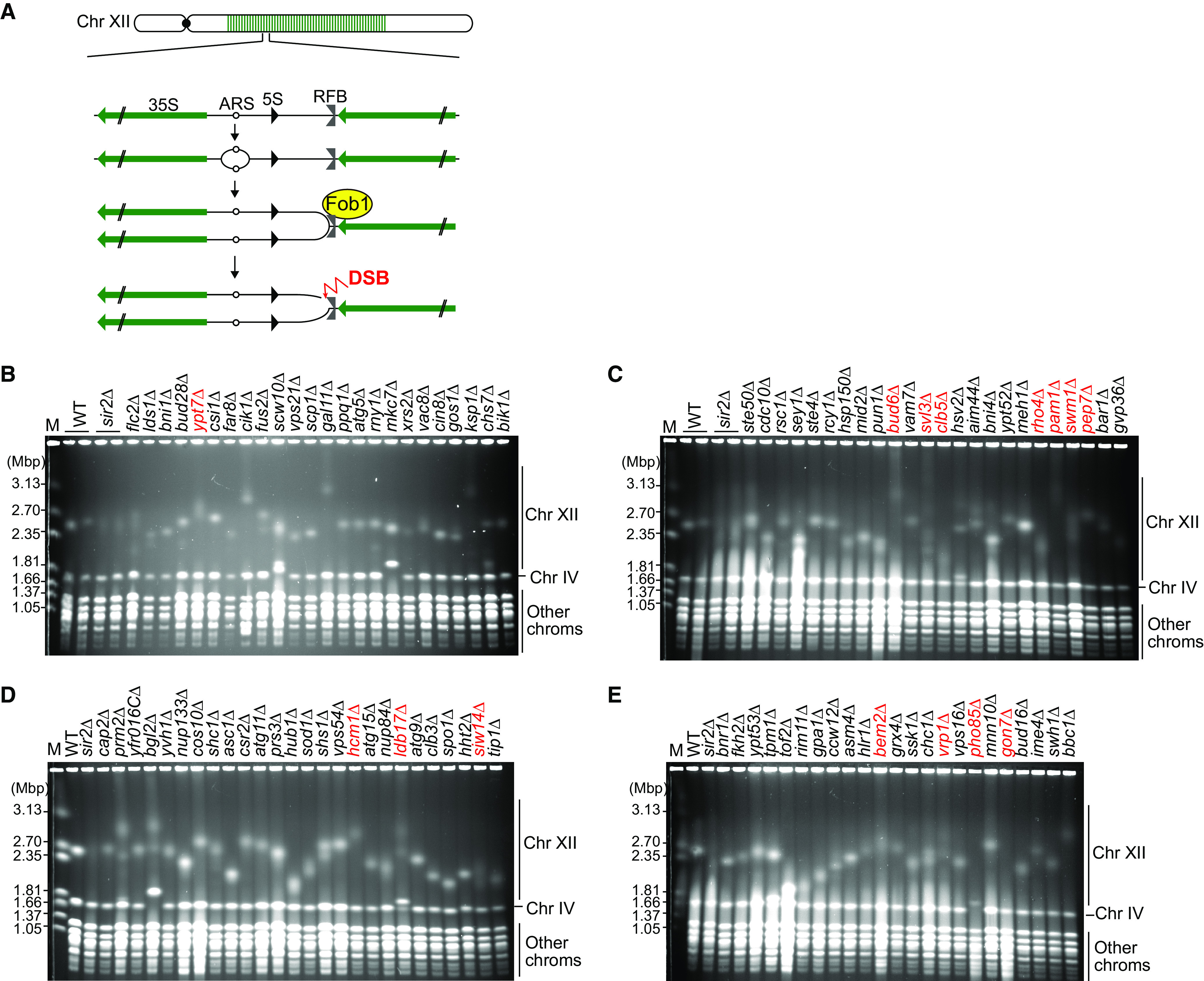
PFGE analysis of rDNA instability in the candidate strains. (A) DNA replication pattern in rDNA and changes in copy number. 35S, 35S rRNA; 5S, 5S rRNA; ARS, autonomously replicating sequence; RFB, replication fork barrier; DSB, DNA double-strand break. (B to E) The indicated mutant strains were patched from a yeast deletion library in which ∼4,800 nonessential genes have been individually disrupted in the BY strain background. Cells were grown from these patches, and genomic DNA was prepared and separated by PFGE. Genomic DNA was also prepared from WT and the *sir2Δ* mutant. DNA was stained with ethidium bromide. M indicates H. wingei chromosomal DNA markers. The mutants subjected to further analysis are indicated in red.

The budding yeast rRNA gene (rDNA) region has a replication fork blocking site and undergoes genome rearrangements in response to replication fork stalling. Therefore, this region is useful for studying genome instability caused by replication-related recombination (5). The rDNA contains a tandem array of ∼150 copies of rDNA sequence at a single locus on chromosome XII (Fig. 1A). Each copy contains 35S and 5S rRNA transcription units, an origin of DNA replication, and a replication fork barrier (RFB) sequence. Although the origin in each copy has the potential to fire, DNA replication is initiated only from a subset of replication origins (6). DNA replication initially proceeds bidirectionally, but the replication fork moving against the 35S rDNA is stalled by Fob1 protein bound to the RFB site (7–9), potentially leading to double-strand break (DSB) formation (10–12) (Fig. 1A). The rDNA copy number frequently changes in a manner mainly dependent on Fob1, suggesting that rDNA instability is induced during the response to replication fork arrest.

We previously performed a genome-wide screen to identify genes that function to maintain the stability of the rDNA region (13). In that screen, we used a collection of mutants in which each of ∼4,800 nonessential genes of budding yeast is deleted, isolated the genomic DNA, and analyzed the size and size heterogeneity of chromosome XII of each mutant by pulsed-field gel electrophoresis (PFGE). The screen identified 708 mutants that showed instability of the rDNA region relative to that of wild-type (WT) cells (13). Many of the genes deleted in these mutants were found to function in nucleic acid transactions such as DNA replication, repair, and recombination. Unexpectedly, however, we also identified genes that had annotated functions in biological processes that seem to be unrelated to maintaining genome stability, such as cell and organelle morphogenesis (13). In this study, we sought to understand how these latter genes contribute to rDNA stability and found that Clb5 is a novel factor that is required for maintaining rDNA stability.

Eukaryotic genomes contain numerous replication origins that have the potential to fire, but only a subset of them are activated to initiate DNA replication in a given S phase (14). The *CLB5* gene and its paralog *CLB6* encode S-phase cyclins, which regulate the timely activation of replication origins in non-rDNA regions in complex with cyclin-dependent kinase. Cells lacking Clb5 show prolonged S phase and defects in origin firing during late S phase, whereas absence of Clb6 has little effect on the duration of S phase or origin firing as long as Clb5 is present (15–18). In the *clb5 clb6* double mutant, entry into S phase is delayed, but the length of S phase and origin firing are restored to WT levels (15–18). These observations suggest that Clb5 and Clb6 both promote timely activation of early firing origins but that only Clb5 is responsible for late origin firing.

Here, we show that rDNA instability in the *clb5*Δ mutant is mostly suppressed when programmed replication fork arrest at the RFB site in rDNA is inhibited by *fob1* mutation. Deletion of the *CLB5* gene resulted in a reduction in the efficiency of replication origin firing in rDNA by half relative to that of WT cells. Both the rDNA instability and origin firing defects in *clb5Δ* cells were suppressed by the additional deletion of *CLB6*. The level of arrested forks was comparable in the tested strains, regardless of the presence or absence of Clb5. In the absence of Clb5, therefore, fewer replication forks initiate DNA replication in the rDNA and stall at the RFB, but these forks are more stably arrested because the arrival of the converging forks is delayed. Although the absence of Clb5 did not influence the level of DSBs or resected DSBs, it resulted in a higher level of recombination intermediates. rDNA instability in *clb5Δ* cells was dependent on Rad52, which is essential for homologous recombination. Thus, persistently arrested forks at the RFB site may lead to DSB-independent, recombination-dependent rDNA instability. Furthermore, absence of Clb5 results in an increase in the distance between active origins due to inefficient replication initiation in rDNA, potentially leading to replication stress that causes damage at non-RFB sites and recombination-mediated rDNA instability.

## RESULTS

### The rDNA region is destabilized in the absence of Clb5.

We previously identified 708 candidate genes that may contribute to maintaining rDNA stability and conducted gene ontology (GO) analysis (13). We have tested the reproducibility of rDNA instability in some mutants lacking genes known to function in nucleic acid metabolism, such as DNA replication, recombination, repair, and transcription (13, 19–22). Among the original 708 rDNA-unstable mutants, however, 113 mutants lack a gene that has an annotated function seemingly unrelated to genome stability but instead is involved in biological processes such as membrane invagination, endocytosis, cytokinesis, nucleus organization, conjugation, cell morphogenesis, cell wall organization or biogenesis, cytoskeleton organization, sporulation, membrane fusion, pseudohyphal growth, invasive growth in response to glucose limitation, and exocytosis or cell budding (13).

In our previous genome-wide screen, we assessed the degree of rDNA stability of each of ∼4,800 mutants only once by PFGE because the number of mutants was too large to analyze in multiplicate. Furthermore, we used a comb with the thinnest teeth to improve throughput, which might have reduced the sharpness of the band and produced false positives, as discussed previously (23). In addition, we now possess an updated version of the original yeast deletion collection. Here, therefore, we first confirmed the reproducibility of rDNA instability in candidate mutants with a GO annotation unrelated to genome stability that were directly obtained from the updated yeast deletion collection. Because 15 of the 113 mutants were analyzed in our previous study (22), and 1 mutant could not be revived from the glycerol stock, the remaining 97 mutants were subjected to confirmation for their rDNA instability.

To assess the rDNA instability, we isolated genomic DNA from the candidate mutants and separated DNA by PFGE using a comb with wider teeth than those of the comb used in our previous screen to improve the sensitivity of our analysis. We then examined the size heterogeneity of chromosome XII, which carries the rDNA array (Fig. 1B to E). The *SIR2* gene encoding a histone deacetylase is known to promote rDNA stability (10, 24, 25); thus, in each gel, we included DNA from WT and the *sir2Δ* mutant, which exhibits an extremely smeared band of chromosome XII compared with that of WT cells (Fig. 1B to E). PFGE analysis showed that the chromosome XII band was prominently smeared in 15 mutants: *ypt7Δ* (Fig. 1B), *bud6Δ*, *svl3Δ*, *clb5Δ*, *rho4Δ*, *pam1Δ*, *swm1Δ*, *pep7Δ* (Fig. 1C), *hcm1Δ*, *ldb17Δ*, *siw14Δ* (Fig. 1D), *bem2Δ*, *vrp1Δ*, *pho85Δ*, and *gon7Δ* (Fig. 1E).

**FIG 2:**
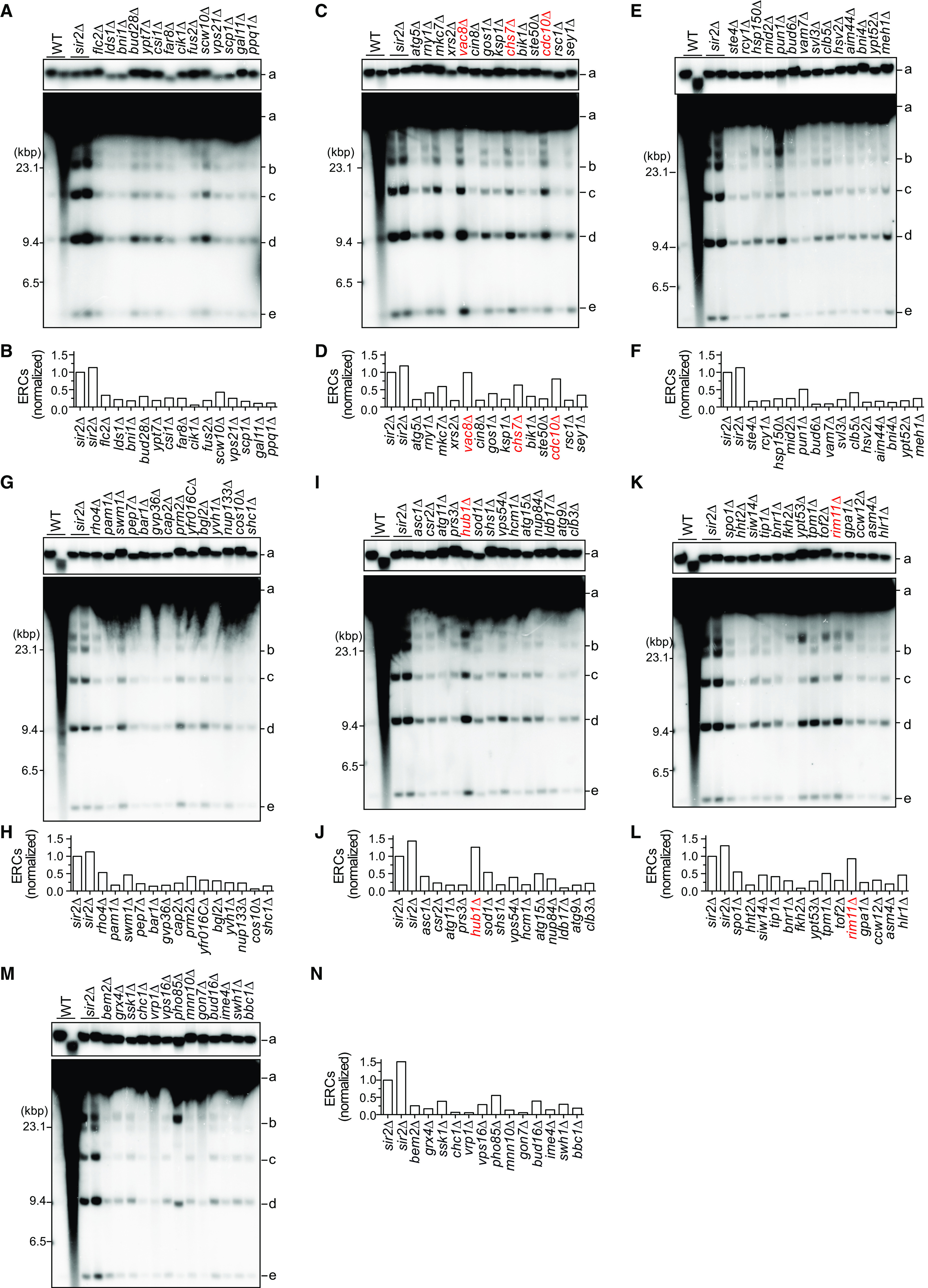
ERC analysis of rDNA instability in the candidate strains. (A, C, E, G, I, K, M) Genomic DNA was prepared from the indicated strains in the yeast deletion library described in the legend to Fig. 1. DNA was also extracted from two independent clones of WT and the *sir2Δ* mutant. DNA was separated by conventional agarose gel electrophoresis, followed by Southern blotting with the rDNA probe. Genomic rDNA (a) and supercoiled and relaxed forms of monomeric and dimeric ERCs (b, c, d, e) are indicated. Sizes of λ DNA-HindIII markers are indicated. (B, D, F, H, J, L, N) Levels of total monomeric and dimeric ERCs relative to those in genomic rDNA. ERC bands were not clearly detected in one WT clone due to poor sample quality, and thus quantification of WT is not shown. The level of ERCs in each mutant was normalized to that in one of the *sir2Δ* samples. The mutants subjected to further analysis are indicated in red.

Changes in rDNA copy number are often associated with the production of extrachromosomal rDNA circles (ERCs), which are excised from genomic arrays. Many of the previously studied rDNA-unstable mutants, such as the *sir2Δ* mutant, accumulate ERCs at a level higher than that of WT cells (24, 25). To examine the level of ERCs in the candidate mutant strains, we separated genomic DNA by conventional agarose gel electrophoresis to resolve ERCs from genomic rDNA and then detected ERCs and genomic rDNA by Southern blotting (Fig. 2). As a reference, genomic DNA samples from WT and the *sir2Δ* mutant were included in the analyses. ERCs were not clearly detected in one of the genomic DNA samples prepared from WT cells due to poor sample quality, and faint ERC bands were detected in the other WT sample (Fig. 2). Therefore, we could not compare the ERC level in each mutant to that in WT cells; instead, we focused our analysis on the novel mutants that exhibited extreme rDNA instability and compared the ERC level in each mutant to that in the *sir2Δ* mutant as a positive control (Fig. 2). The above-mentioned mutants that exhibited a prominently smeared band of chromosome XII in PFGE did not accumulate ERCs to a level comparable to that in the *sir2Δ* mutant (Fig. 1 and 2). Instead, *vac8Δ*, *chs7Δ*, *cdc10Δ* (Fig. 2C and D), *hub1Δ* (Fig. 2I and J), and *rim11Δ* (Fig. 2K and L) were the highest-ranked mutants and produced ERCs at levels comparable to the level in *sir2Δ*, although they did not show severe rDNA instability in PFGE analysis (Fig. 1). Collectively, these analyses enabled us to narrow down the number of genes to test further for confirmation of rDNA instability.

**FIG 3:**
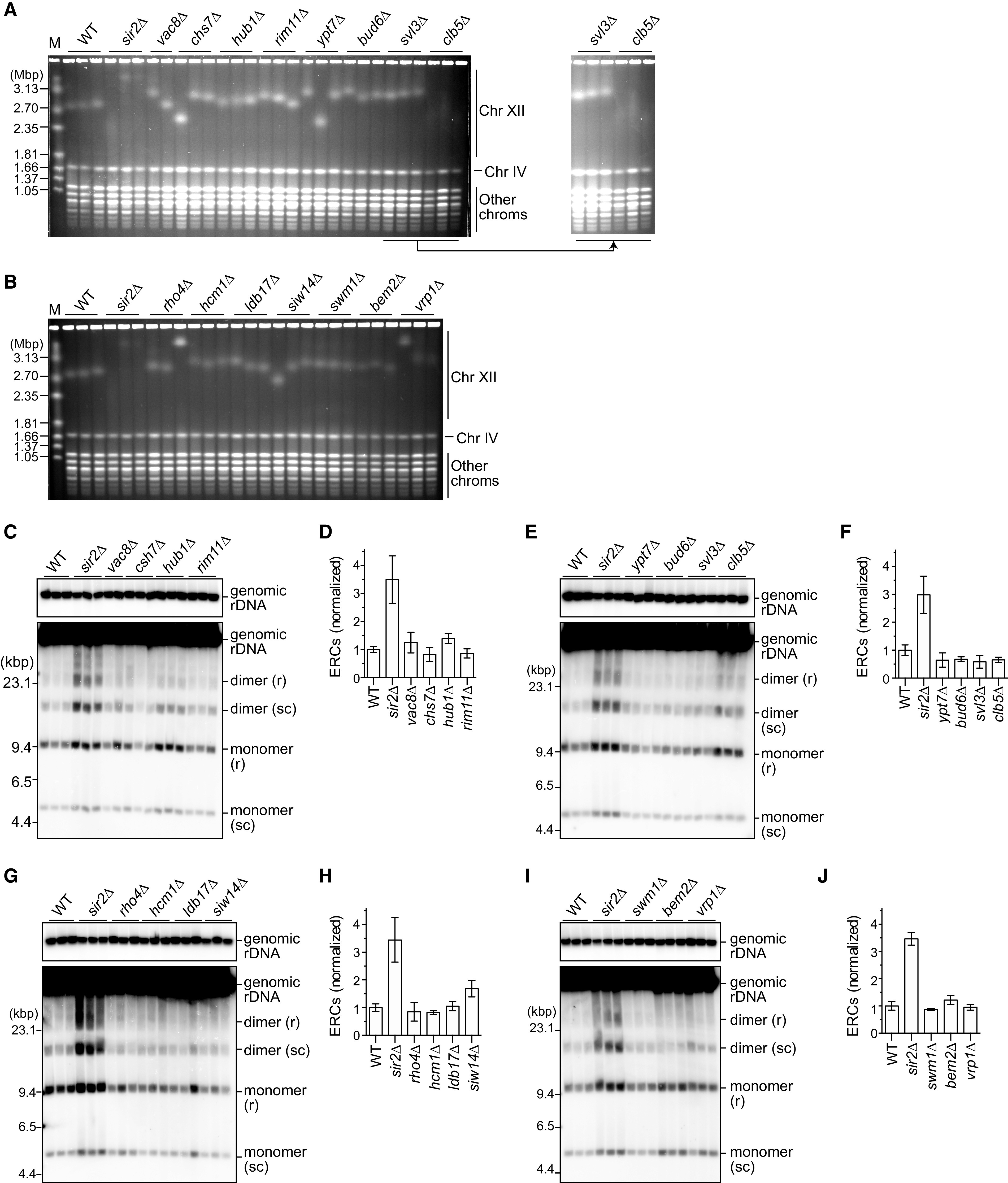
Absence of Clb5 causes rDNA instability. (A, B) PFGE analysis of the size heterogeneity of chromosome XII. The indicated genes were deleted from WT cells in the W303 background. DNA was extracted from three independent clones of WT and all mutant strains except for *vac8Δ* (two clones) and separated by PFGE. DNA was stained with ethidium bromide. M indicates *H. wingei* chromosomal DNA markers. In panel A, the gel image on the right shows part of the gel containing samples from *svl3Δ* and *clb5Δ* mutants, in which the brightness is enhanced for a clearer view of the smeared chromosome XII band in *clb5Δ* mutants. (C, E, G, I) ERC detection. DNA was separated by conventional agarose gel electrophoresis, followed by Southern blotting with the rDNA probe. Genomic rDNA and supercoiled (sc) and relaxed (r) forms of monomeric and dimeric ERCs are indicated. Sizes of λ DNA-HindIII markers are indicated. (D, F, H, J) Level of total monomers and dimers relative to genomic rDNA. The level of ERCs in each mutant was normalized to the average level of ERCs in the WT clones (bars show mean ± standard deviation [SD], except for the bar for *vac8Δ*, which shows the range of two independent clones).

To examine the involvement of these 20 candidate genes in maintaining rDNA stability, we constructed deletion mutants of each gene in the W303 yeast strain background that differs from the BY strain background in which the yeast deletion collection was generated. For unknown reasons, it was not possible to construct mutants lacking *CDC10*, *PAM1*, *PEP7*, *PHO85*, or *GON7*. Among the remaining 15 mutants, only *clb5Δ* showed a smeared band of chromosome XII (Fig. 3A and B). In this mutant, however, the ERC level was similar to that in WT cells (Fig. 3E and F). None of the other 14 mutants produced ERCs at a level comparable to that in the *sir2Δ* mutant (Fig. 3C to J). Taken together, these findings show that absence of Clb5 causes severe rDNA instability in yeast strains of two different genetic backgrounds, demonstrating that Clb5 plays an important role in maintaining rDNA stability.

A previous study showed that the *clb5Δ* mutant exhibits small defects in vacuolar fragmentation under various growth conditions (26). GO analysis thus associates the *CLB5* gene with the biological processes of organelle assembly, organelle fission, and regulation of organelle organization, in addition to its best-known function as an S-phase cyclin required for the activity of CDK, which regulates the initiation and progression of DNA replication (15–18).

### Clb5 suppresses rDNA instability in response to Fob1-mediated replication fork arrest.

**FIG 4:**
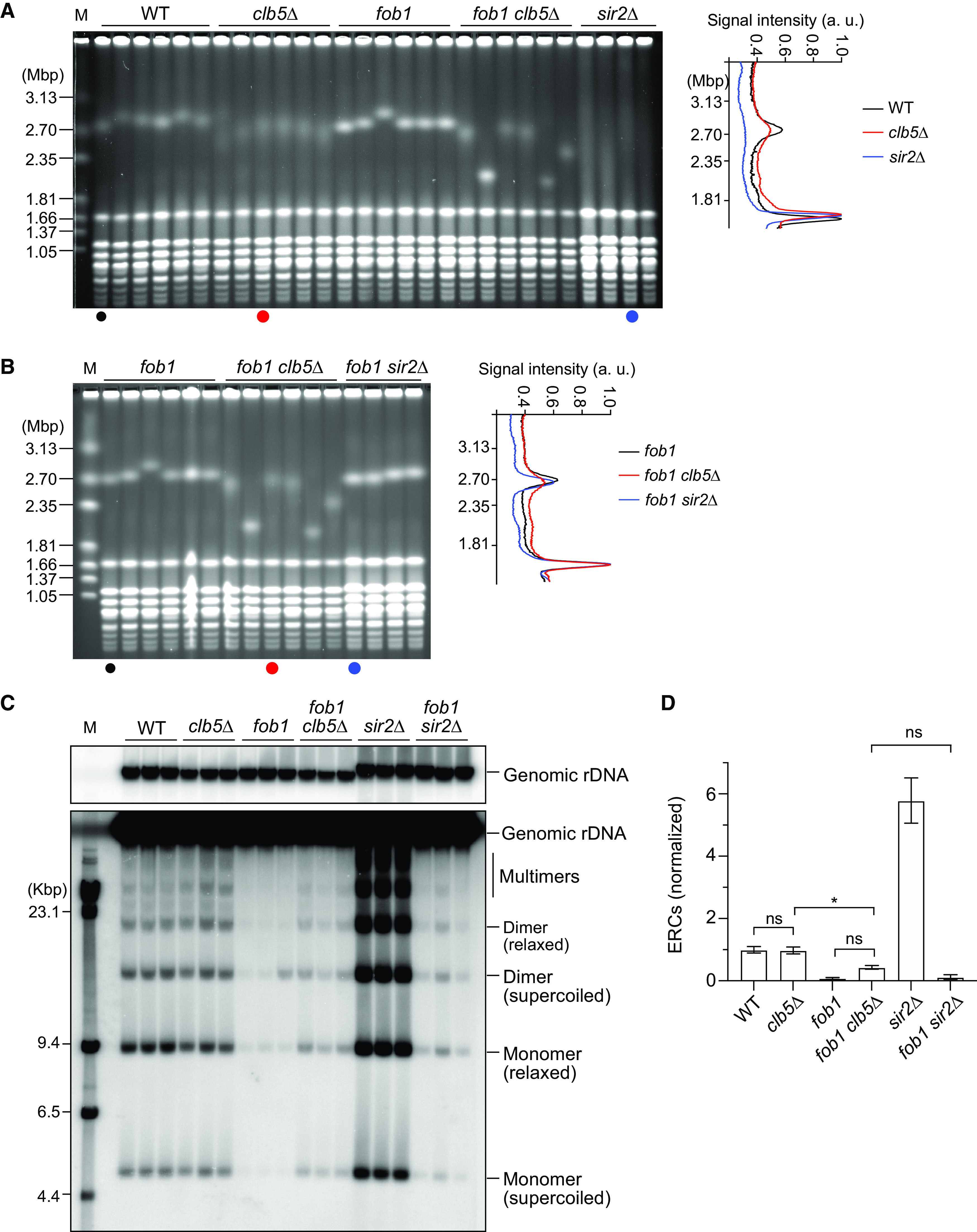
rDNA instability in the *clb5Δ* mutant is mainly dependent on Fob1. (A and B) PFGE analysis of the size heterogeneity of chromosome XII. DNA was extracted from independent clones of the indicated strains and separated by PFGE. DNA was stained with ethidium bromide. M indicates *H. wingei* chromosomal DNA markers. The same DNA samples from the *fob1* and *fob1 clb5Δ* clones were analyzed in panel A and panel B. The graphs on the right show the signal profiles of the indicated lanes. (C) ERC detection. DNA was separated by conventional agarose gel electrophoresis, followed by Southern blotting with the rDNA probe. Genomic rDNA and different forms of ERCs are indicated. M indicates λ DNA-HindIII markers. (D) Quantification of ERCs. The sum of monomers and dimers was determined relative to genomic rDNA, which was normalized to the average of the WT clones (bars show mean ± SD). One-way analysis of variance (ANOVA) was used for multiple comparisons. Asterisks indicate a significant difference at *P* < 0.05; ns indicates no significant difference (*P* > 0.05). a.u., arbitrary units.

To understand how Clb5 promotes rDNA stability, we examined whether rDNA instability in the *clb5Δ* mutant occurs in response to Fob1-mediated replication fork arrest at the RFB. To this end, we constructed a diploid strain heterozygous for *clb5Δ* and *fob1*, induced meiosis, isolated haploid clones by tetrad dissection, and examined rDNA stability in WT, *clb5Δ*, *fob1*, and *fob1 clb5Δ* cells by PFGE (Fig. 4A). To assess the degree of rDNA instability in *clb5Δ* cells, we simultaneously analyzed DNA samples isolated from *sir2Δ* cells in parallel. All six independent *clb5Δ* clones showed smearing of the chromosome XII band compared with the band of WT clones (Fig. 4A). To demonstrate qualitative differences in rDNA stability, the signal intensities in each lane were determined in the region from ∼1.81 to 3.13 Mbp and normalized to the maximum value of signals derived from chromosome IV as a loading control (Fig. 4A, right). The representative *clb5Δ* mutant clone displayed a peak chromosome XII signal with a broader width and lower height compared with that of the WT clone (Fig. 4A, right, black and red lines). The peak of the chromosome XII signal in *sir2Δ*, however, was substantially broader than that in *clb5Δ* (Fig. 4A, right, red and blue lines). Therefore, absence of Clb5 results in rDNA instability but not to the extent seen in the *sir2Δ* mutant.

Smearing of the chromosome XII band in the *clb5Δ* mutant was largely suppressed by the introduction of a *fob1* mutation (Fig. 4A); however, the chromosome XII band in the *fob1 clb5Δ* mutant was still broader than that in the *fob1* single mutant (Fig. 4B, right, black and red lines). This phenotype was not observed for the *sir2Δ* mutant, in which extreme smearing of the chromosome XII band was nearly completely suppressed to the level seen in the *fob1* mutant (Fig. 4B, right, black and blue lines). The partial suppression of rDNA instability by *fob1* mutation is not specific to the *clb5Δ* mutant; we previously reported that *fob1* mutation does not completely suppress rDNA instability in cells lacking Ctf4, a component of the replisome (19). Interestingly, there seemed to be two classes of *fob1 clb5Δ* clones among six independent clones examined, one that showed a broader chromosome XII band compared with that of *fob1* clones (first, third, fourth, and sixth lanes of *fob1 clb5Δ* samples) and another that showed a much sharper chromosome XII band (second and fifth lanes of *fob1 clb5Δ* samples) (Fig. 4B). The *fob1 clb5Δ* clones in the latter class had a smaller chromosome XII band than that of the clones in the former class (Fig. 4B). The biological significance of these two classes is discussed below. Collectively, these results suggest that rDNA instability in the *clb5Δ* mutant mostly depends on Fob1.

We also assessed rDNA instability by ERC analysis (Fig. 4C and D). The *sir2Δ* mutant accumulated ERCs at a level ∼6-fold higher than that in WT cells. Although the *clb5Δ* mutant showed rDNA instability in PFGE, the level of ERCs in the *clb5Δ* mutant was comparable to that in WT (Fig. 4D), as observed in Fig. 3E and F. Thus, unlike many previously characterized rDNA-unstable mutants such as *sir2Δ*, the absence of Clb5 does not cause an accumulation of ERCs. Consistent with our PFGE analysis (Fig. 4A), the level of ERCs was reduced by 2-fold in the *fob1 clb5Δ* mutant relative to that in the *clb5Δ* mutant (Fig. 4C and D); however, the level of ERCs in the *fob1 clb5Δ* mutant was still ∼3- to 5-fold higher than that in the *fob1* and *fob1 sir2Δ* mutants, although these differences were not statistically significant (Fig. 4C and D). Thus, in the absence of Clb5, ERCs are produced predominantly in a Fob1-dependent manner, but some are generated independently of Fob1. Taken together, these results suggest that Clb5 functions to maintain rDNA stability mainly by promoting the proper response to Fob1-mediated replication fork arrest, but it may also prevent the generation of Fob1-independent, rDNA-destabilizing DNA damage in non-RFB regions.

### rDNA instability in the *clb5Δ* mutant is suppressed by deletion of its paralog *CLB6*.

*CLB5* has a paralog, *CLB6*, whose product also acts as an S-phase cyclin. Whereas deletion of *CLB5* causes lengthening of S phase, deletion of *CLB6* has little effect on S-phase duration (16–18). In the *clb5 clb6* double mutant, the onset of S phase is delayed, but the length of S phase is restored to that seen in WT (18). Thus, Clb5 and Clb6 differentially regulate the initiation and progression of S phase.

**FIG 5:**
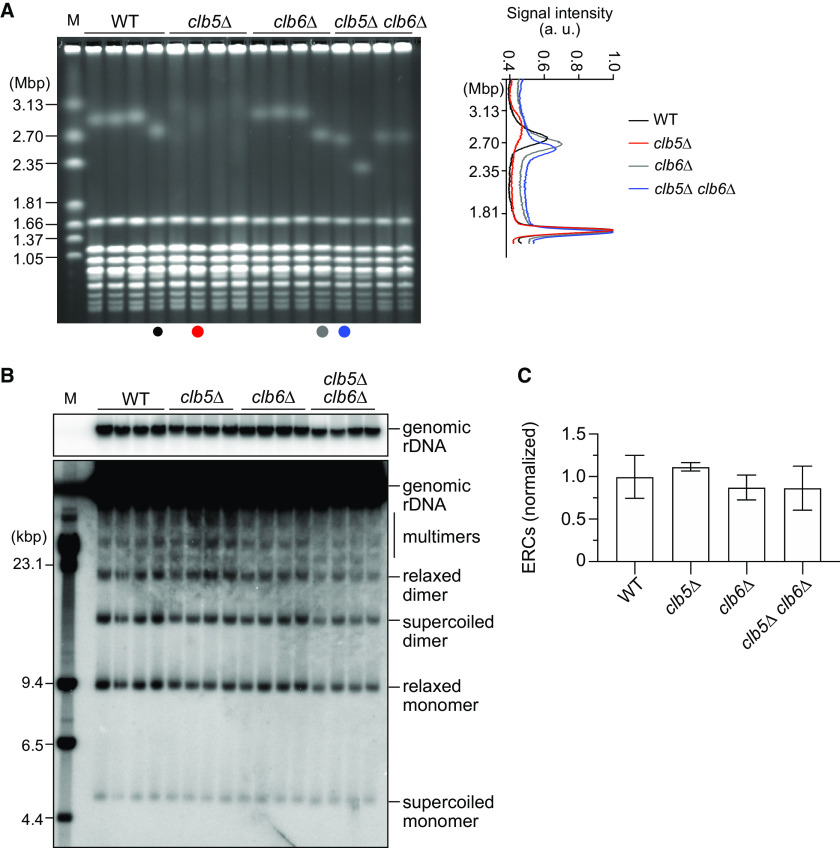
rDNA instability in the *clb5Δ* mutant is suppressed by deletion of *CLB6*. (A) PFGE analysis of the size heterogeneity of chromosome XII. DNA was extracted from independent clones of the indicated strains and separated by PFGE. DNA was stained with ethidium bromide. M indicates *H. wingei* chromosomal DNA markers. The graph on the right shows the signal profiles in the indicated lanes. (B) ERC detection. DNA was extracted by conventional agarose gel electrophoresis, followed by Southern blotting with the rDNA probe. Genomic rDNA and different forms of ERCs are indicated. M indicates λ DNA-HindIII markers. (C) Quantification of ERCs. The sum of monomers and dimers was determined relative to genomic rDNA, which was normalized to the average of WT clones (bars show mean ± SD). One-way ANOVA was used for multiple comparisons. There were no statistically significant differences among the strains.

Next, we sought to understand how Clb5 and Clb6 coordinately regulate rDNA stability. To this end, we constructed a diploid strain heterozygous for *clb5Δ* and *clb6Δ*, induced meiosis, isolated haploid clones by tetrad dissection, and examined rDNA stability in WT, *clb5Δ*, *clb6Δ*, and *clb5Δ clb6Δ* cells. In PFGE, smearing of the chromosome XII band was seen in four independent *clb5Δ* mutant clones examined but not in the *clb6Δ* mutant clones (Fig. 5A). In contrast to the *clb5Δ* single mutant, the *clb5Δ clb6Δ* double mutant showed a homogeneous band of chromosome XII, comparable to that in WT and the *clb6Δ* single mutant (Fig. 5A). Suppression of smearing of the chromosome XII band in the *clb5Δ* mutant by *clb6Δ* was also evident when we compared the shape of the chromosome XII band in the signal profile of the lanes: the *clb5Δ* clone showed a broader and shorter peak of chromosome XII signal relative to that of WT and *clb6Δ*, but the peak became narrower in the *clb5Δ clb6Δ* clone and comparable to that seen in WT and the *clb6Δ* single mutant (Fig. 5A, right). There were no differences in ERC level among WT, *clb5Δ*, *clb6Δ*, and *clb5Δ clb6Δ* cells (Fig. 5B and C). Thus, rDNA instability in the *clb5Δ* mutant is suppressed by deletion of *CLB6*.

### Clb5 regulates replication initiation in the rDNA region.

**FIG 6:**
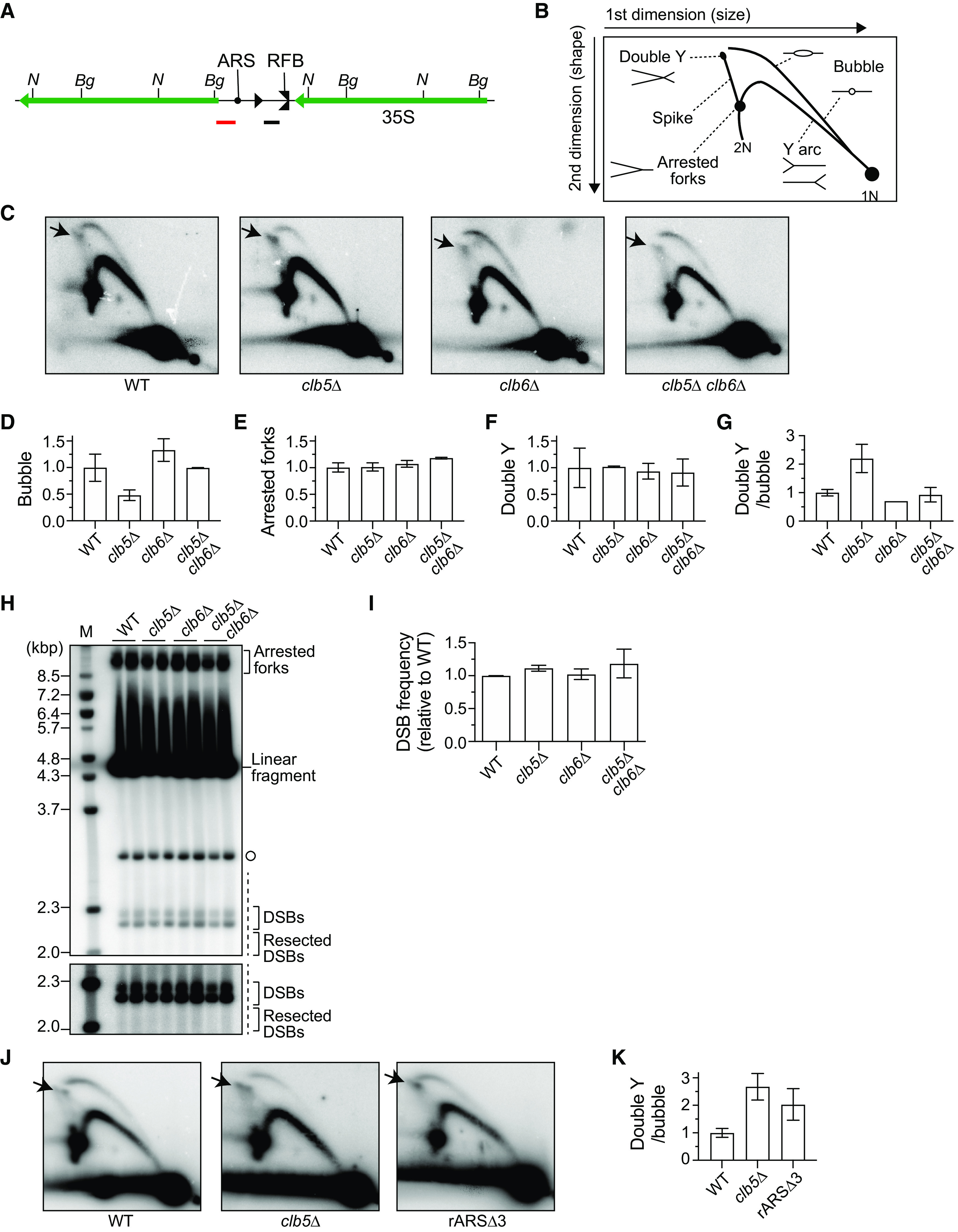
Frequencies of replication initiation, replication fork arrest, and DSBs in *clb5Δ* and *clb6Δ* mutants. (A) Restriction map of the rDNA repeat. Organization of the rDNA repeats is indicated as in Fig. 1A. *N* and *Bg* indicate the sequences recognized by restriction enzymes NheI and BglII, respectively. Black and red bars represent the Southern blotting probes used for 2D and DSB analyses, respectively. (B, C, J) 2D agarose gel electrophoresis. Genomic DNA was digested with NheI. DNA was separated by size in the first dimension and by size and shape in the second dimension and subjected to Southern blotting with the rDNA probe indicated in panel A. The expected migration pattern of different replication intermediates by 2D analysis is shown in panel B. 1N and 2N indicate, respectively, one- and two-unit-length linear DNA molecules. Arrows in panel C and panel J indicate a double Y spot. (D, E, F) Frequency of bubble arcs (D), arrested forks (E), and double Y spots (F) determined by quantifying the signal of each intermediate relative to the total number of replication intermediates. The levels of the different molecules in each mutant were normalized to the average of WT clones (bars indicate the range of two independent experiments). (G, K) Frequency of recombination intermediates determined by the ratio of the double Y spot signal to the bubble arc signal, which was normalized to the average of WT clones. Bars indicate the range of two independent experiments in panel G and the mean ± standard error of the mean (SEM) from three independent samples in panel L. (H) Detection of arrested forks and DSBs. Genomic DNA was digested with BglII and separated by single-dimension agarose gel electrophoresis, followed by Southern blotting with the rDNA probe indicated in panel A. Bands corresponding to arrested forks, linear fragments, DSBs, and resected DSBs are indicated. The open circle represents the terminal fragment containing the telomere-proximal rDNA repeat and its adjacent non-rDNA fragment. The lower panel shows a more exposed contrast image of the phosphorimager signal in the region marked by the dashed line. M indicates λ DNA-BstEII markers. (I) Frequency of DSBs determined as the ratio of DSB signal to arrested fork signal, which was normalized to the average DSB frequency of WT clones (bars indicate ranges of two independent experiments).

Previous studies have demonstrated that Clb5 and Clb6 differentially regulate the timing of replication origin firing in non-rDNA regions; that is, Clb5 and Clb6 both promote firing of early replication origins, but only Clb5 activates late origins (15, 18). Absence of both Clb5 and Clb6 delays entry into S phase but restores the length of S phase and origin firing to WT levels (15–18). To understand how Clb5 and Clb6 regulate replication in the rDNA region, we assessed the frequency of origin firing in this region in the *clb5Δ* and *clb6Δ* mutants (Fig. 6A to D).

To this end, we first grew cells to stationary phase, diluted them into fresh media, and allowed them to grow until they reentered the cell cycle, enabling us to obtain a sample enriched in replicating cells. We then prepared genomic DNA, digested it with the restriction enzyme NheI, which has two recognition sites within each rDNA unit (Fig. 6A), and performed two-dimensional (2D) agarose gel electrophoresis. DNA molecules were separated by molecular mass in the first dimension, followed by mass and shape in the second dimension. DNA was transferred to a nylon membrane and analyzed by Southern blotting with a probe that hybridizes to the NheI fragment containing an origin of DNA replication and an RFB site (Fig. 6A).

In this analysis, DNA molecules where replication is initiated from an origin are detected as bubble-shaped molecules, while those where replication passively progresses through the NheI fragment generate a Y arc (Fig. 6B) (27). In addition, replication forks that are paused at the RFB site within the restriction fragment produce an intensive signal along the Y arc (Fig. 6B). We assessed the frequency of origin firing by determining the proportion of signal for bubble-shaped molecules relative to the total signal for replication intermediates (Fig. 6B and C). The efficiency of origin firing in the *clb5Δ* mutant was lowered by half relative to that of WT (Fig. 6D), demonstrating that Clb5 promotes firing of replication origins in the rDNA. The reduced origin firing observed in the *clb5Δ* mutant was restored in the *clb5Δ clb6Δ* double mutant to almost WT levels (Fig. 6D). Together with the finding that rDNA instability in the *clb5Δ* mutant was suppressed in the *clb5Δ clb6Δ* mutant (Fig. 5), these results indicate that rDNA stability is influenced by the efficiency of replication initiation.

### Replication forks are more stably arrested at the RFB site in the absence of Clb5.

To understand how reduced origin firing in the *clb5Δ* mutant leads to rDNA instability, we examined whether absence of Clb5 influences the frequency of replication fork arrest. More than 90% of replication forks that initiate from replication origins stall at the RFB site (9). Because fewer origins fired in the absence of Clb5 (Fig. 6D), we expected that fewer forks would be stalled at the RFB site in the *clb5Δ* mutant than in WT cells. We assessed the frequency of arrested forks by determining the proportion of signal for arrested fork intermediates relative to the total signal for replication intermediates in our 2D analysis (Fig. 6B and C). The fork blocking activity was comparable among WT, *clb5Δ*, *clb6Δ*, and *clb5Δ clb6Δ* cells (Fig. 6E). This finding implies that, although fewer forks are stalled at the RFB in the *clb5Δ* mutant than in WT cells, these forks are more stably arrested, resulting in a higher level of persistently arrested forks.

Replication fork arrest at the RFB site leads to the formation of DSBs, which are thought to be a major trigger of genome instability (2). It had long been thought that DSBs formed at arrested forks are repaired by homologous recombination; however, our previous study demonstrated that DSBs are rarely resected and their repair does not require recombination proteins in WT cells as long as the cells carry a normal rDNA copy number (19). Thus, changes in rDNA copy number can be dictated by the frequency of DSB end resection.

To determine the frequency of DSBs and resected DSBs formed at the RFB site, we digested DNA with the restriction enzyme BglII and separated it by single-dimension agarose gel electrophoresis, followed by Southern blotting (Fig. 6A and H). We assessed the DSB frequency by determining the ratio of signal for DSBs to that for arrested fork intermediates (Fig. 6H and I). DSB levels were comparable among WT, *clb5Δ*, *clb6Δ*, and *clb5Δ clb6Δ* cells (Fig. 6I). Resected DSBs in the *clb5Δ* mutant were below the detection limit of Southern blotting, but this result indicates that absence of Clb5 does not seem to cause a substantial increase in DSB end resection (Fig. 6H). Thus, Clb5 does not influence the formation or repair of DSBs.

### The *clb5Δ* mutant induces homologous recombination-mediated rDNA instability.

In 2D gel analysis, we also detected replication forks converging from both sides at the RFB, as well as Holliday junction recombination intermediates that are formed during homologous recombination-mediated repair of DSBs (27). Both intermediates generate a double Y spot signal on top of the spike signal and were seen in a similar position in our 2D gel (Fig. 6B and C). As shown in Fig. 6C, the double Y spot signal in the *clb5Δ* mutant seemed to be stronger than that in WT, *clb6Δ*, or *clb5Δ clb6Δ* cells. As a proportion of total replication intermediate signals, however, the double Y spot signal was similar among these cells (Fig. 6F), possibly because more replication intermediates may exist in *clb5Δ*, which has a longer S phase. In contrast, the ratio of double Y spot to bubble signals was clearly increased in the *clb5Δ* mutant (Fig. 6G). Because the arrested fork signal was not elevated (Fig. 6E) and the bubble signal was reduced (Fig. 6D) in *clb5Δ* relative to that in WT, *clb6Δ*, and *clb5Δ clb6Δ* cells, the increased signal ratio of double Y spots in the *clb5Δ* mutant is most probably due to a higher level of recombination intermediates but not converging replication forks. Collectively, these results indicate that recombination frequency is increased in the *clb5*Δ mutant.

We examined whether this elevation of the double Y spot signal is unique to the *clb5Δ* mutant or a similar phenotype is observed in other replication initiation-defective mutants. To this end, we examined the rARSΔ-3 strain, in which all the endogenous rARS sequences lack the ARS consensus sequence III element (28). The rARSΔ-3 strain exhibits a 50% reduction in replication initiation activity compared with that of a strain with the intact rARS sequence (28). We compared the patterns of replication intermediates by performing 2D gel analysis of samples prepared from WT, *clb5Δ*, and rARSΔ-3 cells in parallel. The rARSΔ-3 cells also displayed a strong double Y spot signal compared with that of WT cells (Fig. 6J). The ratio of double Y signal to bubble signal was 2-fold higher in the rARSΔ-3 strain than in WT cells, and this ratio was comparable to or slightly lower than that in the *clb5Δ* mutant, although the differences were not statistically significant (Fig. 6K). These findings raise the possibility that replication initiation defects in the rDNA lead to an accumulation of recombination intermediates.

**FIG 7:**
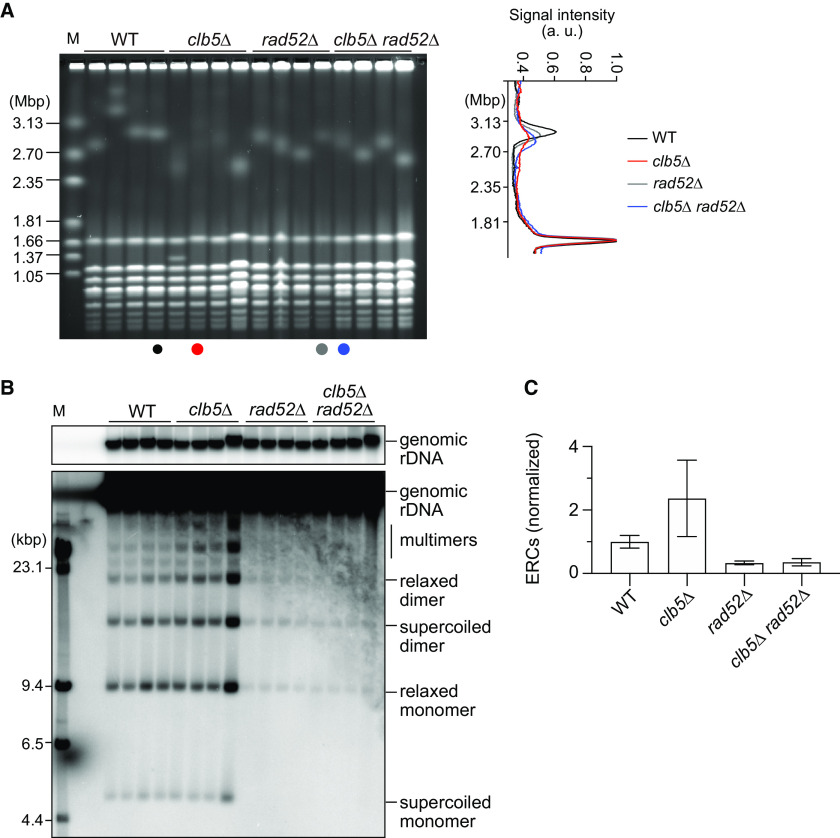
rDNA instability in the *clb5Δ* mutant is suppressed by deletion of *RAD52*. (A) PFGE analysis of the size heterogeneity of chromosome XII. DNA was extracted from independent clones of the indicated strains and separated by PFGE. DNA was stained with ethidium bromide. M indicates *H. wingei* chromosomal DNA markers. The graph on the right shows the signal profiles in the indicated lanes. (B) ERC detection. DNA was extracted by conventional agarose gel electrophoresis, followed by Southern blotting with the rDNA probe. Genomic rDNA and different forms of ERCs are indicated. M indicates λ DNA-HindIII markers. (C) Quantification of ERCs. The sum of monomers and dimers was determined relative to genomic rDNA, which was normalized to the average of WT clones (bars show mean ± SD). One-way ANOVA was used for multiple comparisons.

In PFGE analysis, smearing of the chromosome XII band in the *clb5Δ* mutant was suppressed by deletion of *RAD52*, which is essential for homologous recombination (Fig. 7A). The *clb5Δ* mutant showed an increase in ERCs relative to those in WT cells (Fig. 7B and C); however, the increase was most probably biologically insignificant because this phenotype was not reproducibly observed in other ERC assays (Fig. 3 to 5). Moreover, the fourth sample of the *clb5Δ* clones showed a substantially higher level of ERCs, as well as a sharper chromosome XII band in PFGE, compared with that in the other samples (Fig. 7). Thus, we believe that this clone is most probably an outlier, leading to an artificial increase in the average level of ERCs in the *clb5Δ* mutant in this assay (Fig. 7C). ERC production in the *clb5Δ* mutant was suppressed in the *clb5Δ rad52Δ* mutant to the level seen in the *rad52Δ* mutant (Fig. 7C). These results suggest that homologous recombination-dependent rDNA instability is induced in the *clb5Δ* mutant.

## DISCUSSION

In this study, we confirmed the reproducibility of rDNA instability in 97 previously identified rDNA-unstable mutants that lack genes with a GO annotation that is seemingly unrelated to genome stability, for example, genes functioning in cell and organelle morphogenesis (13). We demonstrated that a mutant lacking the *CLB5* gene exhibits rDNA instability. Based on GO analysis, *CLB5* is associated with the biological processes of organelle fission and regulation of organelle organization, because its deletion causes small defects in vacuolar fragmentation under various growth conditions (26). However, the mechanism by which *CLB5* regulates vacuolar fragmentation remains unknown. The *CLB5* gene and its paralog *CLB6* encode S-phase cyclins required for the activity of Cdc28 and are involved in the initiation and progression of DNA replication (15–18). In this study, we have revealed that Clb5 plays an important role in suppressing homologous recombination-dependent rDNA instability, mainly in response to Fob1-mediated replication fork arrest and partially in response to DNA damage at non-RFB sites, by promoting replication initiation in the rDNA region.

**FIG 8:**
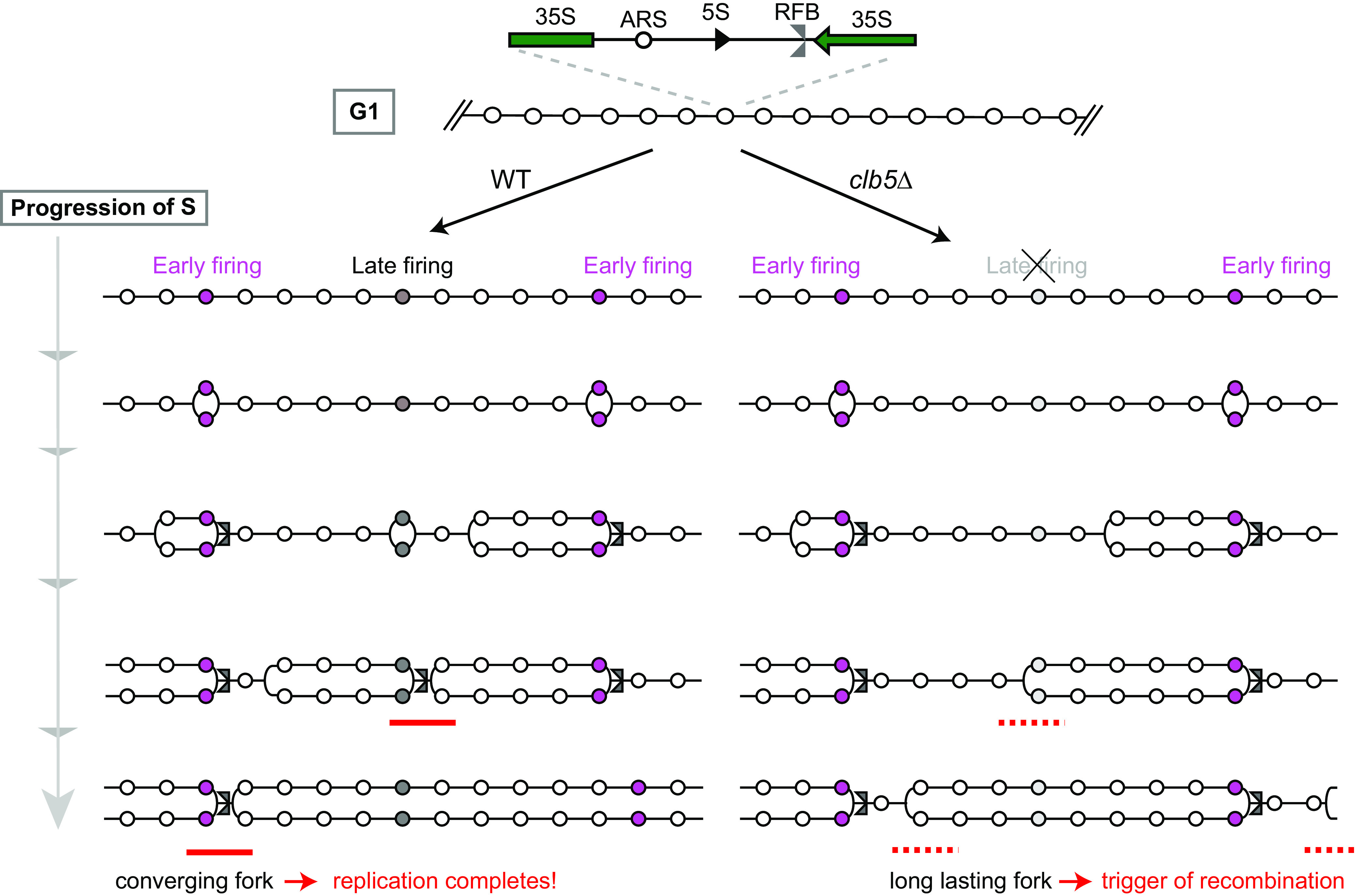
Model of rDNA replication in a replication initiation-reduced condition. (Top) Part of the rDNA in G_1_ phase. Circles represent replication origins (ARS). (Lower left) Progression of DNA replication in WT. The pink-shaded ARSs start replication in early S phase, while the gray-shaded ARSs start late. Rightward moving forks are arrested by the function of Fob1 at the RFB site. About one in five ARSs starts replication in S-phase (6, 34). The red line indicates a converging fork. (Lower right) Progression of DNA replication in the *clb5Δ* mutant. Right-ward moving forks are arrested by the function of Fob1 at the RFB site and remain arrested until the converging fork arrives. Leftward-moving forks have to travel a longer distance because there are fewer late-firing origins. The dashed red line indicates a long-lasting fork. Whereas the number of RFB-arrested forks is similar in WT and *clb5Δ* (six are shown), there are fewer converging forks in the *clb5Δ* mutant. As a result, replication forks in the *clb5Δ* mutant have a longer distance to travel and may encounter problems, such as accidental fork arrest that triggers recombination, on the way.

The initiation of replication across the genome is under temporal control. In non-rDNA regions, Clb5 and Clb6 function redundantly to promote the timely activation of early firing origins, while late origin firing is regulated only by Clb5 because Clb6 is expressed in the early S phase (15, 29). In the rDNA region, each rDNA copy contains a replication origin sequence with the potential to initiate replication, but only a small proportion of these origins fire during rDNA replication (6). We found that deletion of the *CLB5* gene results in a 50% reduction in replication initiation efficiency during exponential growth compared with that of WT cells, whereas deletion of *CLB6* has little effect on replication initiation (Fig. 6C and D). Thus, Clb5 plays a dominant role in the activation of replication origins in the rDNA region. Although we did not examine the timing of origin activation, we speculate that, in the absence of Clb5, late origin firing is defective in the rDNA region (Fig. 8).

We previously demonstrated that replication initiation activity influences rDNA stability (28). When a substantial number of rDNA repeats is lost, cells enter an “rDNA expansion” mode until they regain the normal copy number; this is referred to as “gene amplification” and is thought to compensate for the lost copies of rDNA that are necessary for rRNA production (30). To examine whether replication initiation activity is required for this gene amplification, we constructed an rDNA two-copy strain in which the ARS consensus sequence III was deleted. The resulting rARSΔ-3 strain exhibits lower replication initiation activity in the rDNA region and, relative to the two-copy strain carrying an intact ARS sequence, shows a lower rate of rDNA amplification, suggesting that reduced replication initiation activity is less effective for gene amplification (28). On the other hand, the rARSΔ-3 strain shows ∼6-fold higher frequency of marker gene loss from the rDNA array than that of WT cells. Thus, rARSΔ-3 cells display enhanced rDNA instability in a marker gene loss assay but reduced rDNA instability in gene amplification. The reasons for these discrepancies remain unclear. It is possible that origin firing activity differentially regulates rDNA stability, depending on the number of rDNA repeats carried by the cells; in other words, origin firing activity promotes rDNA instability during gene amplification from an rDNA low-copy-number strain, but efficient origin activity stabilizes rDNA when cells have a normal rDNA copy number. We have not examined the rate of gene amplification for an rDNA low-copy-number strain in the *clb5Δ* mutant; however, our finding that the *clb5Δ* mutant with reduced replication initiation activity shows rDNA instability is consistent with the phenotype of increased marker gene loss seen in the rARSΔ-3 strain.

Replication initiation activity in the rDNA is compromised by mutations in genes encoding components of the origin recognition complex (ORC) and depletion of the Sld2 and Sld3 proteins (31–33). These conditions cause rDNA instability that specifically leads to a reduction in rDNA copy number. Replication initiation defects cause each replication fork to replicate over a longer distance, prolonging S phase to complete rDNA replication. Therefore, it has been suggested that these defects confer a selective advantage on cells with fewer rDNA copies over cells with a normal copy number that have a region of ∼1.3 Mbp to replicate (33). This suggestion may explain our observation that low rDNA copy number strains in the *fob1 clb5* mutant had sharper bands of chromosome XII relative to those of normal rDNA copy number strains (Fig. 4B). The *clb5Δ* cells also showed reduced origin firing activity but exhibited both expansion and contraction of rDNA repeats (Fig. 3–7). We envisage that the efficiency of replication initiation is compromised for both early- and late-firing origins in *orc* mutants and in cells depleted of Sld2 and Sld3. In contrast, deletion of *CLB5* alters the timing of origin activation: early firing origins are activated at the normal time, but late-firing origins are inefficiently activated. The differential impact of mutations on the overall efficiency of origins and replication timing may cause differences in the pattern of rDNA copy number changes, a possibility that should be addressed in future studies.

Previous studies have demonstrated that the prolonged S phase and late origin firing defects in the non-rDNA regions seen in the *clb5Δ* mutant are suppressed in the *clb5Δ clb6Δ* mutant, although the double mutant has delayed entry into S phase (15, 18). It has been proposed that mitotic cyclins may promote S phase in the absence of both Clb5 and Clb6 (15). The rDNA instability and replication initiation defects observed in the *clb5Δ* mutant were also suppressed in the *clb5Δ clb6Δ* mutant (Fig. 5 and 6), thereby suggesting that Clb6 either represses late origin firing or is not sufficient to activate late origins in the rDNA region in the absence of Clb5. These findings demonstrate that the proper activation of late-firing origins may be critical for maintaining rDNA stability.

Our study demonstrates that rDNA instability in the *clb5Δ* mutant occurs in a manner dependent on a homologous recombination protein, Rad52 (Fig. 7). What might be the mechanism underlying the recombination-mediated rDNA instability in the absence of Clb5? Once replication is initiated, the majority of replication forks (>90%) are immediately stalled at the nearest RFB site in a polar fashion (7, 9, 34). Thus, we expected that the *clb5Δ* mutant, which has fewer active origins, would generate fewer forks arrested at the RFB site. Unexpectedly, however, the level of arrested forks in the mutant lacking *CLB5* was comparable to that in WT cells (Fig. 6E), indicating that replication forks are more stably stalled at the RFB in the absence of Clb5. Previous studies have shown that active replication origins in the rDNA are clustered and separated by large gaps without active origins (35–37). A stalled replication fork can be resolved by the arrival of the converging fork proceeding from the nearest origin (3). For the *clb5Δ* mutant, which has fewer firing origins, the converging fork must come from an origin that is further away (Fig. 8). In the absence of Clb5, therefore, more forks remain arrested for a longer time, which is the initiating event for RFB-dependent rDNA instability (Fig. 8).

The replication termination sequence RTS1 induces site-specific fork stalling in fission yeast (1). Previous studies have demonstrated that genome rearrangements are induced by homologous recombination-mediated replication fork restart at stalled forks at the RTS1 site when the arrival of the converging fork is inhibited by the presence of another RTS1 site, but this process does not involve DSB formation (38, 39). It remains unknown whether replication forks arrested at the RFB site in budding yeast rDNA undergo similar genome rearrangements induced by replication fork restart. Nonetheless, absence of Clb5 delays the arrival of the converging fork by impairing the firing of upstream late origins, and as a result, the forks arrested at the RFB site persist longer (Fig. 8). These forks may undergo DSB-independent faulty template switches to restart DNA replication, leading to rDNA instability.

The other consequence of persistent replication fork arrest is the generation of DSBs, which have the potential to induce genome rearrangements when not repaired properly by equal sister chromatid exchange (40). We found, however, that the level of DSBs at the RFB site was similar between cells with Clb5 and cells lacking Clb5 (Fig. 6I). Several factors can influence the outcome of DSB repair. First, one of the intergenic regions of the rDNA unit contains an RNA polymerase II-dependent, bidirectional promoter, named E-pro, upstream of the RFB site (41), and transcription of noncoding RNA from E-pro may induce the dissociation of cohesin from rDNA repeats, leading to unequal sister chromatid recombination that alters the rDNA copy number during DSB repair (10, 41). Thus, Clb5 may suppress rDNA instability by regulating the transcription of noncoding RNA from E-pro and/or cohesin association, two possibilities that need to be examined in future studies. The occurrence of rDNA instability may also depend on the decision of whether cells undergo DSB end resection and repair by homologous recombination (19). In both WT and *clb5Δ*, resected DSBs were below the detection limit of our Southern blotting (Fig. 6H). Therefore, it seems unlikely that absence of Clb5 induces DSB end resection at the RFB.

In our 2D analysis, the double Y spot signal was stronger in the *clb5Δ* mutant than in WT, *clb6Δ*, or *clb5Δ clb6Δ* cells (Fig. 6C). Although we did not see a difference in the ratio of double Y signal to total replication intermediates among these strains, the ratio of double Y signal to bubble arc signal was increased in *clb5Δ* but not in the other strains (Fig. 6G). As mentioned above, this signal most probably corresponds to recombination intermediates. Therefore, DNA damage might occur more frequently in *clb5Δ* than in the other strains, which might explain why only *clb5Δ* shows rDNA instability among the mutants tested. Using 2D analysis, previous studies have analyzed replication intermediates in cells with reduced origin firing efficiency, including the rARSΔ-3 strain, the *orc4*^Y232C^ mutant that mimics the mutation carried by patients with Meier–Gorlin syndrome, and cells depleted of the replication initiation factors Sld2 and Sld3 (28, 32, 33). Those cells all show a substantial reduction in bubble arc signal compared with that of WT cells or a control condition. We analyzed the rARSΔ-3 strain in this study and demonstrated that this mutant also displays an increase in the ratio of double Y signal to bubble arc signal (Fig. 6J and K). Depletion of Sld2 and Sld3 does not lead to an alteration of double Y signals relative to linear molecules, compared with the control condition (32); however, because the bubble arc signal is reduced, the ratio of double Y signal to bubble arc signal is increased in these strains. Whether a similar phenotype is seen for the *orc4*^Y232C^ mutant should be investigated in a future study. Overall, it seems that an increase in recombination intermediates may reflect features associated with reduced origin activity.

What increases the damage in the *clb5Δ* mutant? Because the signals for arrested forks, DSBs, and resected DSBs at the RFB site were not increased in the *clb5Δ* mutant (Fig. 6E, H, and I), we speculate that DNA damage occurs at non-RFB sites in the rDNA region in *clb5Δ*. In fact, although in PFGE the chromosome XII band in the *clb5Δ fob1* double mutant was sharper than that in the *clb5*Δ single mutant, it was still a little broader than that in the *fob1* single mutant (Fig. 4A and B). Moreover, in the ERC assay, more ERCs were detected in the *clb5Δ fob1* double mutant than in the *fob1* mutant (Fig. 4C and D). These results suggest that some *FOB1* (RFB)-independent recombination occurs in *clb5*Δ. One possibility for triggering this recombination is DNA damage caused by the reduced initiation of replication in the *clb5*Δ mutant (Fig. 8). It is known that a longer distance between replication origins induces more genome instability, making a site fragile (42). Moreover, Sanchez et al. observed a broken chromosome XII in the *orc4*^Y232C^ mutant with reduced replication initiation activity (33), although it remains to be determined whether this break occurs during S phase as a consequence of replication problems. It has been speculated that the long-lasting forks have more time to cause problems, such as accidental fork arrest, during the course of travel between origins. A similar situation may occur in the rDNA of the *clb5*Δ mutant. Moreover, a long-lasting fork is expected to make bigger replication bubbles. This might enhance unequal sister chromatid recombination, which would contribute to the rDNA copy number alteration seen in the *clb5Δ* mutant. Further analysis is required to reveal the details of how long-lasting forks cause problems and their resulting DNA damage.

## MATERIALS AND METHODS

### Yeast strains, growth conditions, and genomic DNA preparation.

The mutant strains used in Fig. 1 and 2 are derivatives of the BY4741 background (*MAT***a** *his3Δ1 leu2Δ0 met15Δ0 ura3Δ0*) and obtained from the Yeast Knockout Collection (YSC1053; Open Biosystems [now at Horizon Discovery]) (43–45). The WT strain used in Fig. 1 and 2 was BY4741. The other strains used in this study were derived from NOY408-1b, which is in the W303 background (*MAT***a** *ade2-1 ura3-1 his3-11*,*15 trp1-1 leu2-3*,*112 can1-100*). Mutant strains in which genes of interest were deleted were constructed by a standard one-step gene replacement method, followed by PCR-based genotyping. The mutant strains used in Fig. 3 were constructed by replacing the open reading frame of the gene of interest with the kanMX marker in the NOY408-1b strain. The *sir2Δ* and *fob1 sir2Δ* strains used in Fig. 4 were constructed by replacing the *SIR2* gene with the kanMX marker. The rARSΔ-3 strain used in Fig. 6J and K was TAK209F, which was constructed in a previous study (28). The other haploid strains used in Fig. 4 to 7 were obtained by constructing diploid strains heterozygous for *clb5Δ*::*kanMX* and *fob1*::*LEU2* (Fig. 4), *clb6Δ*::*hphMX* (Fig. 5 and 6), or *rad52Δ*::*hphMX* (Fig. 7), followed by tetrad dissection.

For the PFGE and extrachromosomal rDNA circle (ERC) analyses in Fig. 1 and 2, yeast strains were patched from their glycerol stock onto yeast extract-peptone-dextrose (YPD) plates (1% [wt/vol] yeast extract, 2% [wt/vol] peptone, 2% [wt/vol] glucose, and 2% [wt/vol] agar), and the bulk of cells were grown in 5 ml of YPD medium overnight at 30°C. For other PFGE and ERC analyses, yeast strains were streaked onto YPD plates, a single colony was then inoculated into 5 ml of YPD medium, and cells were grown overnight at 30°C. Cells (5 × 10^7^ cells/plug) were collected and washed twice with 50 mM EDTA (pH 7.5).

For cells subjected to two-dimensional (2D) and DSB analyses, a single colony was inoculated into 5 ml of YPD medium and grown overnight at 30°C until the culture reached the saturation phase of growth. The cells were then inoculated into 100 ml of YPD medium at an optical density at 600 nm (OD_600_) of 0.1 and grown at 30°C until they reached an OD_600_ of 0.4. The cells were immediately treated with 0.1% sodium azide and then collected (5 × 10^7^ cells/plug) and washed twice with 50 mM EDTA (pH 7.5). For PFGE, ERC, 2D, and DSB analyses, genomic DNA was prepared in low-melting-temperature agarose plugs as described previously (19).

### PFGE analysis.

One-third of an agarose plug, along with Hansenula wingei chromosomal DNA markers (Bio-Rad), was separated by electrophoresis on a 1.0% agarose gel (pulsed-field certified agarose, Bio-Rad) in 0.5× Tris-borate-EDTA (TBE) buffer (44.5 mM Tris base, 44.5 mM boric acid, and 1 mM EDTA [pH 8.0]) in a Bio-Rad contour-clamped homogeneous electric field DR-III system using the following conditions: 68 h at 3.0 V/cm, 120° included angle, linear ramp from 300 s of initial switch time to 900 s of final switch time. The gel was stained with 0.5 μg/ml of ethidium bromide and photographed.

### ERC assay.

One-half of an agarose plug, along with 500 ng of lambda HindIII DNA markers, was separated by electrophoresis on a 0.4% agarose gel (15 by 25 cm gel) in 1× Tris-acetate-EDTA (40 mM Tris base, 20 mM acetic acid, and 1 mM EDTA [pH 8.0]) at 1.0 V/cm for ∼48 h at 4°C with buffer circulation in a Sub-cell GT electrophoresis system (Bio-Rad). The buffer was changed every ∼24 h.

DNA was transferred to Hybond-XL (GE Healthcare). Southern blotting was then performed with a probe prepared by PCR amplification of genomic DNA using primers 5′-CATTTCCTATAGTTAACAGGACATGCC and 5′-AATTCGCACTATCCAGCTGCACTC, as described previously (19). The membrane was exposed to phosphor screens for an appropriate amount of time before any signals were saturated, and the radioactive signal was detected using Typhoon FLA 7000 (GE Healthcare). The membrane was reexposed to the phosphor screen for several days and scanned. The scanned images taken after short and long exposures were used to quantify genomic rDNA and ERC bands, respectively, using FUJIFILM Multi Gauge version 2.0 software (Fujifilm). The ratio of ERCs relative to genomic rDNA was determined.

### 2D gel electrophoresis.

2D gel electrophoresis was performed as described previously with slight modifications (46). In brief, one-half of an agarose plug was placed in a 2-ml flat-bottom tube. The plug was equilibrated twice in 1 ml of 1× M buffer (TaKaRa) by rotating the tube for 30 min at room temperature. After discarding the buffer completely, the plug was incubated in 160 μl of 1× M buffer containing 160 units of NheI (TaKaRa) for 7 h at 37°C. The plug and 600 ng of lambda HindIII DNA markers were separated by electrophoresis on a 0.4% agarose gel (SeaKem Agarose LE; Lonza) in 1× TBE buffer at 1.32 V/cm for 14 h at room temperature with buffer circulation in a Sub-cell GT electrophoresis system (15 by 20 cm gel; Bio-Rad). The gel was stained with 1× TBE buffer containing 0.3 μg/ml of ethidium bromide and photographed. Gel slices containing DNA ranging from 4.7 to 9.4 kb were excised, rotated 90°, and cast in a 1.2% agarose gel (SeaKem Agarose LE; Lonza) containing 0.3 μg/ml of ethidium bromide in 1× TBE. The second-dimension gel electrophoresis was performed in 1× TBE buffer containing 0.3 μg/ml of ethidium bromide at 6.0 V/cm for 5 h at 4°C with buffer circulation in a Sub-cell GT electrophoresis system (Bio-Rad). DNA was transferred to Hybond-XL (GE Healthcare).

Southern blotting was performed with a probe prepared by PCR amplification of genomic DNA using primers 5′-CATTTCCTATAGTTAACAGGACATGCC and 5′-AATTCGCACTATCCAGCTGCACTC, as described previously (19). The membrane was exposed to phosphor screens for several days and the radioactive signal was detected using Typhoon FLA 7000 (GE Healthcare). ImageJ (NIH) was used to quantify bubbles, Y arcs containing RFB spots, RFB spots, and double Y spots.

### DSB assay.

The DSB assay was performed as described previously (19). In brief, one-third of an agarose plug was placed in a 2-ml flat-bottom tube. The plug was equilibrated four times in 1 ml of 1× Tris-EDTA (TE; 10 mM Tris base [pH 7.5] and 1 mM EDTA [pH 8.0]) by rotating the tube for 15 min at room temperature. The plug was then equilibrated twice in 1 ml of 1× NEBuffer 3.1 (New England Biolabs) by rotating the tube for 30 min at room temperature. After discarding the buffer completely, the plug was incubated in 160 μl of 1× NEBuffer 3.1 buffer containing 160 units of BglII (New England Biolabs) overnight at 37°C. The plug and 600 ng of lambda BstEII DNA markers were separated by electrophoresis on a 0.7% agarose gel (15 by 20 cm gel) in 1× TBE buffer at 2.0 V/cm for 21 h at room temperature with buffer circulation in a Sub-cell GT electrophoresis system (Bio-Rad). The gel was stained with 0.5 μg/ml of ethidium bromide.

DNA was transferred to Hybond-XL. Southern blotting was performed with a probe prepared by PCR amplification of genomic DNA using primers 5′-ACGAACGACAAGCCTACTCG and 5′-AAAAGGTGCGGAAATGGCTG, as described previously (19). The membrane was exposed to phosphor screens overnight. The radioactive signal was detected using Typhoon FLA 7000 (GE Healthcare). Signals of DSBs and arrested forks were quantified using FUJIFILM Multi Gauge version 2.0 software (Fujifilm). The ratio of DSBs relative to arrested forks was determined and normalized to the average value of WT samples.

### Statistical analysis.

Statistical analysis was performed by using GraphPad Prism software (version 8.0).
